# The REM Sleep Behavior Disorder Screening Questionnaire is not Valid in De Novo Parkinson's Disease

**DOI:** 10.1002/mdc3.12591

**Published:** 2018-03-01

**Authors:** Claire Halsband, Antonia Zapf, Friederike Sixel‐Döring, Claudia Trenkwalder, Brit Mollenhauer

**Affiliations:** ^1^ Department of Clinical Neurophysiology University Medical Center Göttingen Germany; ^2^ Department of Neurosurgery University Medical Center Göttingen Germany; ^3^ Department of Medical Statistics University Medical Center Göttingen Germany; ^4^ Paracelsus‐Elena‐Klinik Kassel Germany; ^5^ Department of Neurology University Medical Center Göttingen Germany

**Keywords:** DeNoPa cohort, prodromal marker, Parkinson's disease, REM sleep behavior disorder screening questionnaire, RBDSQ

## Abstract

**Background:**

Rapid eye movement (REM) sleep behavior disorder (RBD) is one of the most specific prodromal indicators for Parkinson's disease (PD).

**Objectives:**

To test the validity of the RBD‐Screening Questionnaire (RBDSQ) in assessing RBD in early PD.

**Methods:**

The RBDSQ was completed before video‐supported polysomnography (vPSG) by 134 de novo PD patients, 109 matched controls without neurological disorder (CTR) and 30 subjects with idiopathic RBD (iRBD) without clinical signs of PD; results were compared with vPSG‐confirmed RBD diagnosis.

**Results and Conclusions:**

Sensitivity/specificity of the RBDSQ for the PD cohort were 0.44/0.84 at the previously published cut‐off score of 6 for PD patients, and the area under the curve (AUC) 0.68 (95% CI, 0.56–0.79). By contrast, in the iRBD/CTR cohort the RBDSQ (cut‐off = 5) had a sensitivity/specificity of 0.97/0.84 and an AUC of 0.95 (95% CI, 0.90–1.00). Subanalysis of question 6 only (4 subitems asking for dream enactment) at a cut‐off score of 1 revealed a sensitivity of 0.74 and a specificity of 0.70 for the de novo PD cohort, AUC was 0.74 (95% CI, 0.63–0.84). RBDSQ is an insufficient screening tool for RBD in de novo PD. New screening tools for RBD assessment need to be developed.

## Introduction

Rapid eye movement (REM) sleep behavior disorder (RBD) is characterized by abnormal behavior, often qualified as dream enactment, during REM sleep. It is accompanied by a loss of physiological muscle atonia during REM sleep recorded on electromyography (EMG) (REM sleep without atonia RWA).^1^ RBD is considered the most specific prodromal marker for neurodegenerative diseases associated with the misprocessing of α‐synuclein (i.e., Lewy body dementia, multiple system atrophy and Parkinson's disease [PD]). Up to 25% of de novo PD patients have been shown to have RBD,^2^ and prevalence increases with disease duration.^3^, ^4^ As video‐supported polysomnography (vPSG) for the diagnosis of RBD is costly and not readily available, a valid screening tool for facilitating identification of populations at risk of developing α‐synuclein aggregation disorders is of the utmost importance for clinical trials with putative neuroprotective agents.^5^ However, depending on the population screened, the RBD‐screening questionnaire (RBDSQ) has shown disparate results in performance^6^, ^7^, ^8^ (see table 3); a validation for RBD‐screening in de novo and prodromal PD has not yet been undertaken. In this study, we validated the RBDSQ with polysomnographic results in a de novo PD cohort and assessed the validity of the RBDSQ for use in future studies in early PD. Results of the RBDSQ in a cohort of idiopathic RBD (iRBD) subjects and controls (CTR) are compared to the de novo PD cohort results. A subanalysis of sensitivity and specificity at a cut‐off score of 1 for question 6 only of the RBDSQ (subitems 6.1, 6.2, 6.3 and 6.4) is added.

## Methods

### Participants

The analyzed data were extracted from the baseline evaluation of the DeNoPa cohort, a prospective longitudinal single‐center observational cohort study of patients with de novo PD and age‐ and sex‐matched neurologically healthy controls (for further details see,^9^ recruitment period 2010–2011). CTR were excluded if they reported a sleep disorder at initial presentation.^2^ A separate cohort of 30 iRBD patients with polysomnography‐verified RBD‐diagnosis according to the criteria mentioned below was added for further evaluation (recruitment period 2012–2016).

All participants gave their full written informed consent for participation in the study as well as for video‐polysomnography as described.^3^, ^9^ The local Ethical Committee approved the project (Approval no. FF89/2008).

### Polysomnography

All subjects underwent a cardiorespiratory video‐supported PSG (Xltec: Excel Tech Ltd) according to American Academy of Sleep Medicine (AASM) criteria on two consecutive nights; RBD was diagnosed according to criteria established by Schenck et al.^10^ and the International Classification of Sleep Disorders, 2nd edition (ICSD 2).^11^ For details, see Sixel‐Doring et al.^2^, ^12^

REM without atonia was quantified by surface EMG activity of the mentalis muscle during REM sleep according to the method published by the SINBAR group and the cut‐off value for 100% specificity was set at a mentalis EMG activity rate of 18.2%.^23^

For diagnosis of RBD, study subjects needed to show REM associated motor behavior and/or vocalizations plus an EMG activity rate above the specific cut‐off. Subjects with REM associated motor behavior and/or vocalizations but EMG activity below the specific cut‐off were counted as RBD negative. Application of the new ICSD 3^1^ diagnostic criteria for RBD to the polysomnography measurements did not result in a different classification of RBD positive and negative subjects in comparison to the ICSD 2 criteria.

Patients and controls completed the RBDSQ without assistance as part of a routine work‐up before undergoing polysomnography and without prior information about RBD.

### RBD‐Screening Questionnaire

The RBDSQ consists of 10 questions with 13 items overall; items 3, 6.1, 6.2, and 6.3 focus on dream enactment behavior, item 10 asks about central nervous system (CNS) disease. In the original validation study^8^ a cut‐off score of 5 was defined in a heterogeneous RBD cohort. Nomura suggested a cut‐off score of 6 for PD patients.^13^

### Statistical Analysis

The RBDSQ answer profiles of the PD patients were compared to polysomnography results. The same procedure was applied to iRBD patients and CTR. Mean and standard deviations for metric demographic and clinical data were calculated if a normal distribution was present and compared via student t test for independent samples (with Satterthwaite approximation if variances were unequal). For variables with non‐normal distribution the median and interquartile range (IQR = 1^st^ quartile to 3^rd^ quartile) were calculated and compared via the Mann‐Whitney U test. Categorical variables are presented in absolute and relative frequencies and were compared via chi‐squared test or Fisher's exact test (in case of cell frequencies < 5). Associated p values are for descriptive purposes and not for evaluation of significance. We measured test performance of the whole RBDSQ by plotting a receiver operating characteristic curve (ROC) and calculating the respective area under the curve (AUC) with its two‐sided 95% confidence interval (CI). The same statistical procedures were applied for a subanalysis of the four subitems of question 6 of the RBDSQ. For comparison, the AUC and its 95% CI for question 6 of the Parkinson's Disease Sleep Scale‐2 (PDSS‐2) were calculated.^14^, ^15^ Reliability of the RBDSQ was assessed by calculating Cronbach's α.

## Results

By vPSG we identified 33 RBD positive de novo PD patients of the DeNoPa cohort, that were compared to the 101 RBD negative ones. Furthermore, 30 iRBD patients were compared to 107 vPSG confirmed RBD negative CTR.

Comparison of demographic and clinical data in the different groups is summarized in Table 1.

**Table 1:** Demographic and Clinical Data

Variable	PD, RBD+ (n = 33)	PD, RBD – (n = 101)	p‐value PD RBD+ vs. PD RBD‐	iRBD (n = 30)	CTR (n = 107)	p‐value iRBD vs. CTR
Age (mean +/− SD)	66 +/− 8.2	64 +/− 10.2	0.18	65 +/− 11.2	65 +/− 6.9	0.93
Sex male (%)	22 (67%)	66 (65%)	0.89	18 (60%)	64 (60%)	0.96
PD duration in months median (IQR)	12 (8–24)	17 (12–24)	0.36	n.a.	n.a.	n.a.
MDS‐UPDRS score median (IQR)	35 (24–47.5)	34 (23–48.5)	0.8	17 (13–25)	2 (1–5)	0.00
RBDSQ score median (IQR)	4.2 (2.2–8)	2.4 (1.1–4.3)	0.003	9 (7–11)	2 (1–4)	0.00
PDSS‐2 question 6 score median (IQR) mean +/− SD	1 (0–2) 0.97 +/− 1.16	0 (0–1) 0.68 +/− 0.98	0.19 0.20	1 (0–2.25) 1.30 +/− 1.37	0 (0–1) 0.50 +/− 0.75	0.002 0.004

Abbreviations: PD, Parkinson's disease; RBD, REM sleep behavior disorder; CTR, controls; iRBD, idiopathic RBD patients; IQR, interquartile range; MDS‐UPDRS, Movement Disorder Society‐Unified Parkinson's Disease Rating Scale; RBDSQ, RBD screening questionnaire; PDSS, Parkinson's Disease Sleep Scale.

No relevant differences in age or sex were noted between the RBD positive and negative PD groups (*p =* 0.18 and *p =* 0.89). RBDSQ scores were higher in the RBD positive PD group (4.2 versus 2.4, *p =* 0.003).

**Figure 1:**
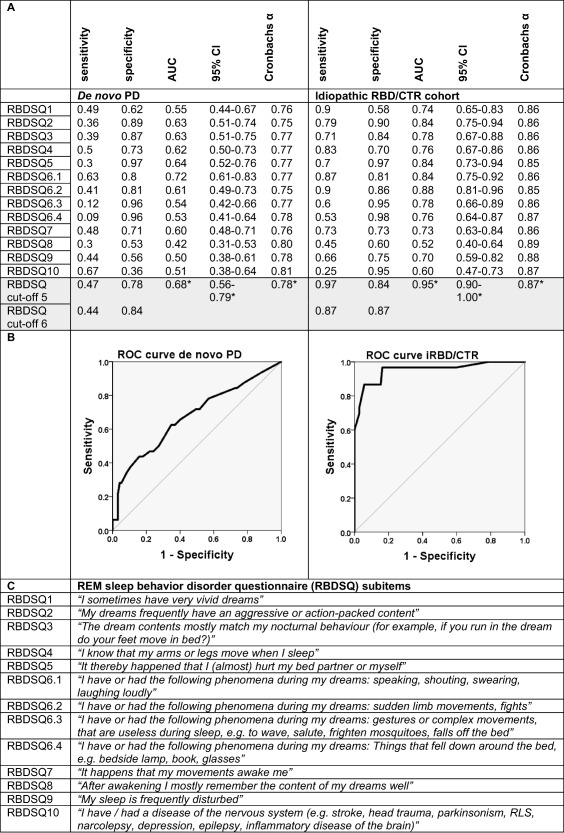
(**A**) RBDSQ performance in a de novo PD cohort compared to idiopathic RBD patients/ controls; (**B**) Corresponding Receiver Operating Characteristic (ROC) curves; (**C**) RBDSQ subitems. Abbreviations: AUC, area under the curve; CI, confidence interval. *Indicated cut‐off not applicable

In the PD group the ROC curve revealed an AUC of 0.68 (95% CI, 0.56–0.79) with a sensitivity of 0.44 and a specificity of 0.84 at a cut‐off score of 6 (0.47 and 0.78, respectively at a cut‐off score of 5). Looking at the individual items the maximal sensitivity for RBD in PD was 0.67 for item no. 10 (for wording of the question see figure 1) and 0.63 for item 6.1. Specificity showed maximum values of 0.97 for item 5, and 0.96 for items 6.3 and 6.4. Cronbach's α was 0.78 for the overall questionnaire including items 1–10; rising to a maximum of 0.81 by omitting item 10 (answered positive for all PD patients).

Additional analysis of the 30 iRBD patients in comparison to CTR (without RBD) revealed an AUC of 0.95 (95% CI, 0.90–1.0), a sensitivity of 0.87 and a specificity of 0.87 at cut‐off score of 6 (0.97 and 0.84, respectively at a cut‐off score of 5). Maximal sensitivity was 0.9 for items 1 and 6.2. Maximal specificity was 0.98 for item 6.4, 0.97 for item 5 and 0.95 for items 6.3 and 10.

Subanalysis of RBDSQ question 6 only with the subitems 6.1, 6.2, 6.3, and 6.4 gave an overall AUC of 0.74 (95% CI, 0.63–0.84), a sensitivity of 0.74 and a specificity of 0.70 for the de novo PD cohort at a cut‐off score of 1. For the iRBD/CTR cohort AUC was 0.96 (95% CI, 0.93–0.99), sensitivity 1.00, and specificity 0.74 if at least one of the subitem questions was answered positive (cut‐off score of 1; see table 2).

**Table 2:** Subanalysis of Sensitivity/Specificity of RBDSQ Question 6 Only

	*De novo* PD					Idiopathic RBD/CTR cohort				
	Sensitivity	Specificity	AUC	95% CI	Cronbachs α	Sensitivity	Specificity	AUC	95% CI	Cronbachs α
RBDSQ6.1	0.63	0.8	0.72	0.61–0.83	0.59	0.87	0.81	0.84	0.75–0.92	0.74
RBDSQ6.2	0.41	0.81	0.61	0.49–0.73	0.56	0.9	0.86	0.88	0.81–0.96	0.70
RBDSQ6.3	0.12	0.96	0.54	0.42–0.66	0.54	0.6	0.95	0.78	0.66–0.89	0.75
RBDSQ6.4	0.09	0.96	0.53	0.41–0.64	0.58	0.53	0.98	0.76	0.64–0.87	0.78
RBDSQ6 cut‐off 1	0.74	0.70	0.74^a^	0.63–0.84^a^	0.64^a^	1.00	0.74	0.96^a^	0.93–0.99^a^	0.80^a^

Abbreviations: AUC, area under the curve; CI, confidence interval.

^a^Indicated cut‐off not applicable

**Table 3:** RBDSQ Validations in PD Cohorts at a Cut‐Off Score of 6 in the Literature

	Sensitivity	Specificity	AUC	95% CI
*Bolitho et al. 2014* 21	0.80^a^	0.55^a^		
	0.74^b^	0.68^b^		
*Nomura et al. 2011* 13	0.84	0.96	0.95	
*Chahine et al. 2013* 22	0.74	0.93	0.8	0.7–0.9
*Stiasny‐Kolster et al. 2015* 20	0.64^c^	0.68^c^	0.67^c^	0.54–0.80^c^
	0.78^d^	1.00^d^	0.95^d^	0.90–1.00^d^
Li et al. 2017^e,^, 6	0.77	0.88	0.85	0.82–0.88
Our results (*de novo* PD)	0.44	0.84	0.68	0.56–0.79

Abbreviations: AUC, area under the curve; CI, confidence interval.

^a^polysomnography using REM atonia index

^b^polysomnography using REM EMG density

^c^RBDSQ prior to clinical interview

^d^post clinical interview

^e^meta‐analysis presenting pooled estimates of RBDSQ performance

Answer profile of the RBDSQ and PDSS‐2‐question 6: Did you suffer from distressing dream at night? (Answers: very often, 4; often, 3; sometimes, 2; occasionally, 1; never, 0) were compared. Spearman's rank correlation coefficient was 0.53 (95%CI, 0.41–0.65) for the PD cohort and of 0.51 (95% CI, 0.36–0.63) for the iRBD/CTR cohort. The AUC was 0.57 (95% CI, 0.45–0.69) and sensitivity/specificity of PDSS‐2 question 6 at a cut‐off score of 2 was 0.30/0.80 for the PD cohort and 0.67 (95% CI, 0.55–0.79) and 0.33/0.92 for the iRBD/CTR cohort respectively.

## Discussion

Based on our results, the RBDSQ seems not suitable for screening for RBD in early PD.

For the early PD cohort, RBDSQ compared to the gold standard vPSG for diagnosing RBD only reached a maximal sensitivity of 0.47 when cut‐off scores of 5 and 6 were applied. Lowering the RBDSQ cut‐off score to 1 provides a sensitivity of 0.88, but would result in a reduced and inacceptable specificity of 0.22.

However, a better sensitivity is reached by only including subitems 6.1 to 6.4 of question 6 of the RBDSQ. At a cut‐off score of 1, sensitivity increases to 0.74 at a reasonable specificity of 0.70 in the de novo PD cohort. Thus, using question 6 only for screening for RBD in de novo PD might be more appropriate. Previous studies, including the original paper of Stiasny‐Kolster et al.,^8^ focused on the most specific questions 6.3, 6.4, and 5, using those in our de novo PD cohort would indeed result in a high specificity of 0.91 but at the expense of sensitivity being reduced to 0.34.

Using the RBDSQ as a screening tool in the general population (e.g., for identifying subjects at risk of neurodegenerative disease), we would expect lower positive predictive values because of the even lower frequency of RBD that is assumed in the general population. Thus, calculating the positive predictive value at the cut‐off score of 6 gives a value of 0.48 for our de novo cohort and a value of 0.33 for the mixed de novo/CTR cohort (0.42 and 0.28 respectively at a cut‐off score 5; data not shown).

In contrast, the RBDSQ performed well in our cohort with clinically suspected, and afterwards polysomnography‐proven, iRBD. As PD diagnosis accounts for one positive answer in the RBDSQ, the cut‐off score of 6 is best compared to a cut‐off score of 5 in iRBD. At the cut‐off score of 5 the sensitivity reached 0.97 combined with a good specificity of 0.84. This is comparable or even better than in previous validation studies with iRBD patients. In 2016, Marelli et al. found a high sensitivity of 0.97 but a low specificity of 0.46 at a cut‐off score of 5.^16^ The lower specificity may be due to inclusion of patients with various sleep disorders whereas our comparison group only included subjects who were carefully screened for the absence of sleep related complaints (CTR). Lee et al. compared the Korean RBDSQ results of iRBD patients to CTR and reached a sensitivity of 0.89 and a specificity of 0.98 at a cut‐off score of 4.5.^17^ Our results do show good performance in iRBD patients referred to a sleep laboratory for clinically suspected, and therefore probably more violent RBD. However, more subtle types of dream enactment are seldomly reported in clinical history and thus limit the performance of the RBDSQ, as these would only be diagnosed by population‐wide polysomnography.

Low performance of the RBDSQ in our de novo PD cohort might be due to several factors. In a cohort of de novo PD we found a subtler RBD phenotype with typical jerky movements of the extremities and few vocalizations in REM sleep.^2^, ^18^ These findings are in line with the maximum sensitivity detected in question 6.1 related to shouting (etc.) in sleep. However, the first RBDSQ‐validation study in 2007 that reported a sensitivity of 0.96 and a specificity of 0.56 at a cut‐off score of 5 was mainly completed in a cohort of violent RBD phenotypes and included iRBD and narcolepsy patients.^8^ The correlation between an increased RBDSQ‐score and more violent RBD has been confirmed in several studies.^13^, ^19^

Second, the RBDSQ was applied in a routine work‐up without prior sleep assessment, thus mimicking the situation in a population screening where no specific information on RBD is given prior to screening. Stiasny‐Kolster et al. reported a markedly enhanced sensitivity of the RBDSQ when prior sleep assessment with information about RBD took place.^20^

Other validated RBD‐scales also focus on violent dream enactment and therefore are not expected to be more sensitive. We also assessed question 6 of the PDSS‐2 at a cut‐off score of 2 for detection of RBD in de novo PD. This showed a similar specificity to the RBDSQ at a cut‐off score of 6 but an even lower sensitivity of 0.30.

Low sensitivity of the RBDSQ is probably not due to the questionnaire itself but to the nature of RBD and its diagnostic criteria. Questionnaires in general might not be appropriate for diagnosis of more subtle RBD forms as patients are often not aware of their sleep disorder, especially if no bedpartner is available.

Even if only about 25% of de novo PD patients have RBD,^2^ it is one of the most specific predictors we have for identifying high‐risk individuals for α‐synuclein aggregation disorders in the population. For diagnosing RBD in de novo PD and in emerging population‐based cohorts better screening tools need to be developed and assessed. A focus might be the development of portable and easy‐to‐apply polysomnography or actigraphy measurements.

## Authors' Roles

1. Research Project: A. Conception, B. Organization, C. Execution; 2. Statistical Analysis: A. Design, B. Execution, C. Review and Critique; 3. Manuscript Preparation: A. Writing the First Draft, B. Review and Critique.

C.H.: 1A, 1B, 1C, 2B, 2C, 3A, 3B, 3C

A.Z.: 2A, 2B, 2C

F.S‐D.: 1C, 3B

T.C.: 1A, 1B, 3C

B.M.: 1A, 1B, 1C, 3C

## Disclosures

**Ethical Compliance Statement**: We confirm that we have read the Journal's position on issues involved in ethical publication and affirm that this work is consistent with those guidelines. All co‐authors have been substantially involved in the study. No undisclosed groups or persons have had a primary role in the study or manuscript preparation. All co‐authors have seen and approved the submitted version of the paper, accept responsibility for its content, and agree to the order of author names.

**Financial Sources and Conflict of Interest**: This project has received funding from the European Union's Horizon 2020 research and innovation program Propag‐Ageing under grant agreement no. 634821. The DeNoPa cohort study was supported by unrestricted grants from the University Medical Centre Göttingen, the Paracelsus‐Elena‐Klinik, Kassel, Germany, the Michael J Fox Foundation for Parkinson's Research (MJFF), and from TEVA Pharma.

**Financial Disclosures for the preceding 12 months**: C.H.'s work has been supported by a research grant of the European Union (Propag‐Ageing, Horizon 2020 program). She declares no conflict of interest concerning the article submitted.

A.Z. declares that there are no conflicts of interest relevant to this work.

F.S‐D. has received honoraria for speaking engagements from UCB, Grünenthal, AbbVie, and Medtronic. Congress participation was sponsored by Abbvie. She declares no conflicts of interest concerning the article submitted. C.T. has received honoraria for consultancy from Britannia, UCB, Abbvie, Benevolent, Novartis, Orion and speaker's honoraria from UCB, Vifor and Grünenthal. C.T. has received grants from the Michael J. Fox Foundation for Parkinson's Research and the European Union (Propag‐Ageing, Horizon 2020 program). CT has intellectual property rights as an author of a book for PD patients (Schattauer publisher) and of PDSS‐2 scale. B.M. has received independent research grants from TEVA‐Pharma, Desitin, Boehringer Ingelheim, GE Healthcare and honoraria for consultancy from Bayer Schering Pharma AG, Roche, AbbVie, TEVA‐Pharma, Biogen, and for presentations from GlaxoSmithKline, Orion Pharma, TEVA‐Pharma and travel costs from TEVA‐Pharma. B.M. is member of the executive steering committee of the Parkinson Progression Marker Initiative and PI of the Systemic Synuclein Sampling Study of the Michael J. Fox Foundation for Parkinson's Research and has received grants from the Deutsche Forschungsgemeinschaft (DFG), BMBF, EU (Horizon2020), Parkinson Fonds Deutschland, Deutsche Parkinson Vereinigung, Michael J. Fox Foundation for Parkinson's Research, Stifterverband für die deutsche Wissenschaft, and has scientific collaborations with Roche, Bristol Myers Squibb, Ely Lilly, Covance/BioLegend, and Biogen.
